# Natural product celastrol suppressed macrophage M1 polarization against inflammation in diet-induced obese mice via regulating Nrf2/HO-1, MAP kinase and NF-κB pathways

**DOI:** 10.18632/aging.101302

**Published:** 2017-10-16

**Authors:** Dan Luo, Yumeng Guo, Yuanyuan Cheng, Jia Zhao, Yu Wang, Jianhui Rong

**Affiliations:** ^1^ School of Chinese Medicine, Li Ka Shing Faculty of Medicine, The University of Hong Kong, Pokfulam, Hong Kong; ^2^ Department of Pharmacology and Pharmacy, Li Ka Shing Faculty of Medicine, The University of Hong Kong, Pokfulam, Hong Kong

**Keywords:** obesity, inflammation, adipose tissue, liver, macrophage polarization, celastrol

## Abstract

Macrophage polarization is implicated in the inflammation in obesity. The aim of the present study was to examine the anti-inflammatory activities of botanical triterpene celastrol against diet-induced obesity. We treated diet-induced obese C57BL/6N male mice with celastrol (5, 7.5 mg/kg/d) for 3 weeks, and investigated macrophage M1/M2 polarization in adipose and hepatic tissues. Celastrol reduced fat accumulation and ameliorated glucose tolerance and insulin sensitivity. Celastrol down-regulated the mRNA levels of macrophage M1 biomarkers (e.g., IL-6, IL-1β, TNF-α, iNOS) in cell culture and in mice. The underlying mechanisms were investigated in murine macrophage RAW264.7 cells. Our results demonstrated that celastrol might control macrophage polarization through modulating the cross-talk between the following three mechanisms: 1) suppressing LPS-induced activation of MAP kinases (e.g., ERK1/2, p38, JNK) in a concentration dependent manner; 2) attenuating LPS-induced nuclear translocation of NF-κB p65 subunit in a time dependent manner; 3) activating Nrf2 and subsequently inducing HO-1 expression. HO-1 inhibitor SnPP diminished the inhibitory effects of celastrol on the activation of NF-κB pathway and the pro-inflammatory M1 macrophage polarization. Taken together, celastrol exhibited anti-obesity effects via suppressing pro-inflammatory M1 macrophage polarization. Thus, our results provide new evidence for the potential of celastrol in the treatment of obesity.

## INTRODUCTION

Obesity is recognized as a state of chronic low-grade inflammation and associated with type-2 diabetes, dyslipidemia, nonalcoholic fatty liver disease, atherosclerosis, hypertension and acute myocardial infarction [1-3]. Obesity-induced inflammation appears to occur mainly in insulin-sensitive tissues (e.g., adipose tissue, liver and muscle) in obese humans, especially those with symptomatic metabolic disorders [4]. As for the proinflammatory mediators, previous studies detected enormous numbers of proinflammatory macro-phages and neutrophils in adipose and hepatic tissues from animal models and obese patients [5]. Macrophages are well-known to undergo classical M1 polarization or alternative M2 polarization. M1 macrophages secrete pro-inflammatory cytokines such as interleukin-6 (IL-6), tumor necrosis factor-α (TNF-α), interleukin-1β (IL-1β) [4]. Adipose tissue inflammation mainly stimulates M1 macrophage polarization and even induces the phenotypic switch from M2 to M1 macrophages [6]. M1 macrophages release the pro-inflammatory cytokines to exacerbate inflammation in adipose tissues and promote insulin resistance in obesity [7, 8]. On the other hand, obesity drives adipose tissues to undergo pathological expansion, thereby creating hypoxic microenvironment [9]. A recent study found that hypoxia affected ∼1,300 genes including leptin, adiponectin, IL-1β and IL-6 [10]. Pro-inflammatory cytokines synergistically alter the cell-cell communica-tions between macrophages, preadipocytes and adipocytes, causing insulin resistance and type-2 diabetes [8, 11]. Interestingly, M1 macrophages may communicate with the remote cells via several pro-inflammatory cytokines, for example, colonic M1 macrophages cause inflammation and insulin resistance in “remote” adipose tissues [12]. It was previously underestimated that hepatic macrophages could directly cause insulin resistance. Liver is densely populated with resident macrophages, also known as Kupffer cells [13]. M1 macrophages may also promote inflammation in adipose and hepatic tissues. For the exact mechanisms by which hepatic macrophages cause insulin resistance in liver, pro-inflammatory cytokines and free fatty acids are well-known to induce the over-activation of c-JUN N-terminal kinase (JNK), leading to the insulin resistance and diabetes [14]. Along this line, M1 macrophages could promote insulin resistance via activating JNK and nuclear factor-kappa B (NF-κB) pathways. Indeed, JNK and NF-κB pathways are often activated in multiple tissues and thereby mediate tissue inflammation in obesity. On the other hand, M2 macrophages overexpress anti-inflammatory factors such as interleukin-10 (IL-10) and arginase-1to ameliorate insulin resistance in obesity [6, 15]. M2 Kupffer cells ameliorated insulin resistance and delayed the progression of obesity-induced steatohepatitis in mice [16]. Therefore, we postulate that macrophage polarization should be timely reprogrammed towards an anti-inflammatory M2 phenotype for resolution of inflammation in obesity.

Triterpene celastrol was initially identified from the plant *Tripterygium wilfordii* for its anti-oxidant, anti-inflammatory, anti-cancer and anti-rheumatoid arthritis activities [17, 18]. Celastrol was recently identified from more than 1000 small molecules as the best hit for treating diet-induced obesity [19]. It was found that celastrol might enhance leptin activity for suppressing appetite and inducing weight loss [19, 20]. Indeed, the pathological disruption of the leptin-adiponectin axis contributed to increased systemic inflammation and oxidative stress in patients with the metabolic syndrome (MS) [21]. However, celastrol may elicit anti-obesity activities by several other mechanisms. One earlier study showed that celastrol increased antioxidant capacity and improved lipid metabolism in diet-induced obesity [22]. Another recent study demonstrated that celastrol improved obesity and metabolic dysfunctions through activating heat shock factor (HSF)-PGC1α transcriptional axis [23]. Presumably, celastrol could regulate the anti-inflammatory activity, leptin activity and food intake in a coordinated manner. Nevertheless, it remains elusive whether celastrol fights obesity-induced inflammation through skewing macrophage polarization from proinflammatory M1 to anti-inflammatory M2.

In the present study, we treated diet-induced obese mice with celastrol for three weeks and confirmed the effects of celastrol on fat accumulation, glucose tolerance and insulin sensitivity. We hypothesized that celastrol might promote weight loss in diet-induced obese mice via attenuating inflammation. Thereby, we examined not only the effects of celastrol on the expression of proinflammatory biomarkers in adipose tissue and liver but also the activation of inflammation-related signaling pathways including MAP kinases, NF-κB, nuclear factor erythroid-2-related factor/heme oxygenase-1 (Nrf2/HO-1) pathways in macrophages. In addition, we explored whether celastrol could skew macrophage polarization from proinflammatory M1 toward anti-inflammatory M2 phenotype.

## RESULTS

### Celastrol promoted weight loss and glucose homeostasis

**Figure 1:**
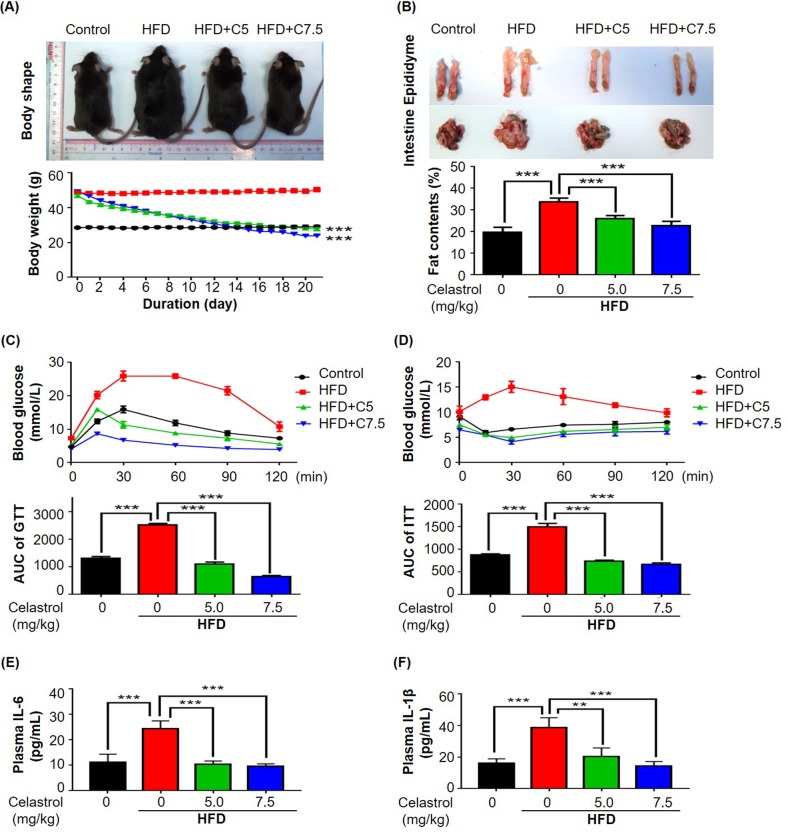
Celastrol attenuated diet-induced obesity in C57BL/6N mice (**A**) Celastrol promoted weight loss. Diet-induced obese mice were treated with celastrol (0, 5, 7.5 mg/kg/d) for 21 days, whereas control mice were fed with normal diet. Body weight was daily measured. (**B**) NMR determination of fat contents. After 21-day treatment, fat contents in mice were analyzed by Bruker minispec NMR analyzer. (**C**) Glucose tolerance test (GTT). After glucose injection, blood was collected and analyzed for glucose level. The area under curve (AUC) for each group was calculated. (**D**) Insulin tolerance test (ITT). After insulin injection, blood was collected and analyzed for glucose level. The AUC for each group was calculated. (**E**) Plasma level of IL-6. After 21-day treatment, plasma was isolated from mouse blood and measured by commercial mouse IL-6 ELISA kit. N=3; HFD, HFD only; C5, celastrol (5 mg/kg/d); C7.5, celastrol (7.5 mg/kg/d). (**F**) Plasma level of IL-1β. After 21-day treatment, plasma was isolated from mouse blood and measured by commercial mouse IL-1β ELISA kit. N=3; HFD, HFD only; C5, celastrol (5 mg/kg/d); C7.5, celastrol (7.5 mg/kg/d); **, *p* < 0.01; ***, *p* < 0.001

To examine the potential of celastrol to treat diet-induced weight gain, we firstly generate a mouse model of over-weight by feeding mice with 60 kcal % high fat diet (HFD) over three months. We subsequently divided the obese mice into three treatment groups: HFD, on HFD only; HFD+C5, on HFD supplemented with 5 mg/kg/d celastrol; and HFD+C7.5, on HFD supplement-ed with 7.5 mg/kg/d celastrol. Celastrol treatment was continued for another three weeks while the wellness and body weight of mice were monitored on daily basis. No signs of intoxication were detected in behavioral assessment and hematoxylin and eosin (H&E) staining of major organs (data not shown). As shown in Fig. 1A, celastrol at the doses of 5 and 7.5 mg/kg/d effectively promoted weight loss to the control levels after two weeks although the animals were still on HFD. Mice in HFD group did not gain body weight during the treatment period. Fat mass was visually examined and accurately determined by NMR technology at the end of celastrol treatment. As shown in Fig. 1B, celastrol reduced fat accumulation in HFD mice in a dose-dependent manner (n = 10, *p* < 0.001). Especially, celastrol at the dose of 7.5 mg/kg/d recover the fat contents in HFD mice to the similar level in control mice. To further determine the effects of celastrol on glucose tolerance and insulin sensitivity, we conducted two common experimentrs, intra-peritoneal glucose tolerance test (ipGTT) and insulin tolerance test (ITT), respectively. As shown in Fig. 1C and 1D, mice in HFD group evidently showed aberrantly high blood glucose level, known as prediabetic state. Interestingly, HFD mice treated with celastrol at the doses of 5 and 7.5 mg/kg/d effectively consumed blood glucose to the similar level in control mice (n = 6, *p* < 0.001). Similarly, mice in HFD group became much less sensitive to exogenous insulin and maintained high blood glucose level. Celastrol at the doses of 5 and 7.5 mg/kg/d effectively improved insulin sensitivity in HFD mice (n = 6, *p* < 0.001). Celastrol-treated and control mice exhibited similar blood glucose level in response to insulin injection.

### Celastrol reduced the plasma levels of IL-6 and IL-1β in mice

To evaluate the *in vivo* effects of celastrol on the release of pro-inflammation cytokines, mouse plasma was collected from mice in four treatment groups (i.e., Control, HFD, HFD+C5, HFD+C7.5). The plasma levels of IL-6, TNF-α and IL-1β were determined by enzyme-linked immunosorbent assay (ELISA) kits. As shown in Fig. 1E and 1F, HFD increased the plasma levels of IL-6 and IL-1β while celastrol effectively reduced the plasma levels of IL-6 and IL-1β to the normal levels in a dose-dependent manner. No signals were detected for TNF-α in mouse plasma.

### Celastrol suppressed pro-inflammatory macrophage M1 polarization and slightly enhanced macrophage M2 polarization in adipose tissue and liver

**Figure 2:**
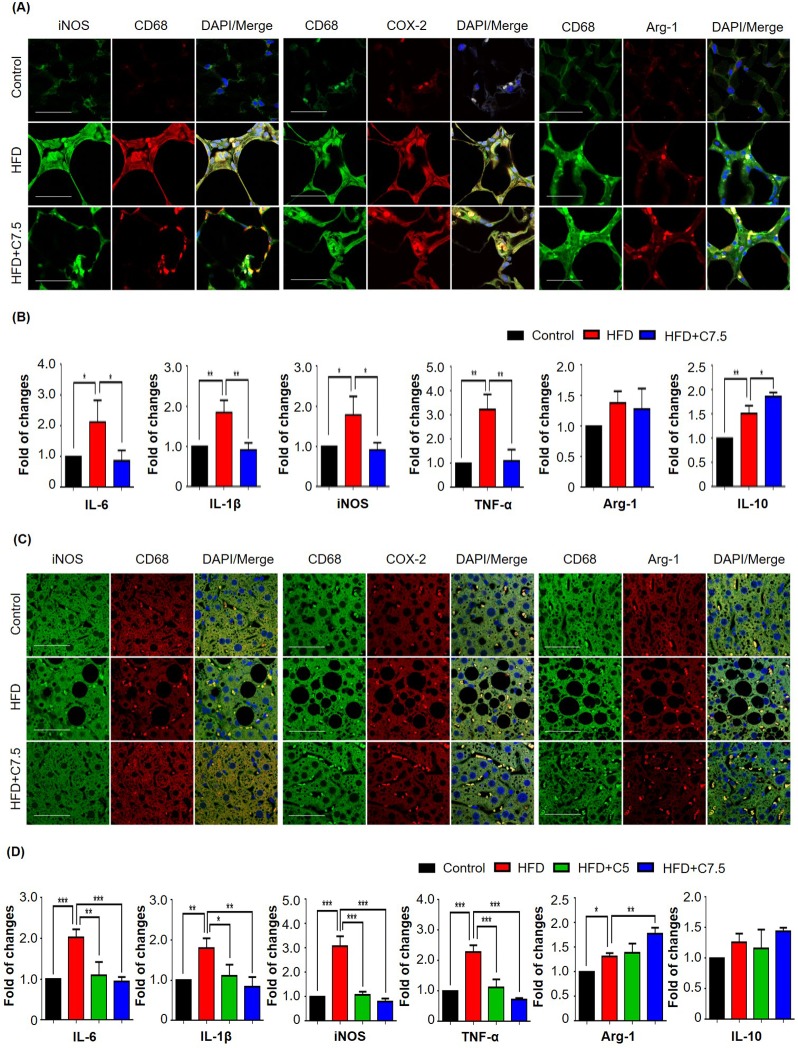
Celastrol differentially affected the expression of macrophage M1 and M2 biomarkers in epididymal adipose tissues and liver (**A**) Detection of iNOS, COX-2, and arginase-1 in epididymal adipose tissues. After 21-day treatment, epididymal fat pads were recovered from mice, and stained with specific antibodies. CD68 was stained as pan-macrophage bomarker whereas the cell nuclei were stained with DAPI. The sections were imaged under a Zeiss LSM 780 confocal microscopy. Representative images were shown. Scale bar, 50 μm. (**B**) qRT-PCR quantification of macrophage M1 and M2 biomarkers in epididymal adipose tissues. Total RNAs were extracted from adipose tissues and analyzed by qRT-PCR technique using QuantiTect SYBR Green PCR Kit and specific DNA primers from Qiagen. N = 3; HFD, HFD only; C7.5, celastrol (7.5 mg/kg/d). (**C**) Detection of iNOS, COX-2, and arginase-1 in liver tissues. Livers were recovered from mice, and stained with specific antibodies. CD68 was stained as pan-macrophage biomarker whereas the cell nuclei were stained with DAPI. The sections were imaged under a Zeiss LSM 780 confocal microscopy. Representative images were shown. Scale bar, 50 μm. (**D**) qRT-PCR quantification of macrophage M1 and M2 biomarkers in liver tissues. Total RNAs were extracted from livers and analyzed by qRT-PCR technique using QuantiTect SYBR Green PCR Kit and specific DNA primers from Qiagen company. N = 3; HFD, HFD only; C5, celastrol (5 mg/kg/d); C7.5, celastrol (7.5 mg/kg/d); *, *p* < 0.05; **, *p* < 0.01; ***, *p* < 0.001.

To determine the *in vivo* effects of celastrol on macrophage polarization, mouse epididymal fat pads and livers were recovered from animals in three treatment groups (i.e., Control, HFD, HFD+C7.5), and subsequently examined the expression of the typical biomarkers for inflammation and macrophage polarization by immunostaining and qRT-PCR techni-ques. For adipose tissues, as shown in Fig. 2A, epididymal fat pads were stained with antibodies against inducible nitric oxide synthase (iNOS), cyclooxygenase-2 (COX-2) and arginase-1, whereas CD68 was stained as a pan macrophage biomarker, and DAPI as a probe for cell nuclei. HFD increased the expression of iNOS and COX-2 in macrophages, while celastrol selectively reduced the expression of iNOS over COX-2. Interestingly, celastrol somehow enhanced the expression of arginase-1 in macrophages. To further examine the effects of celastrol on macrophage M1/M2 polarization in adipose tissues, we determined the expression levels of the typical macrophage biomarkers by qRT-PCR. As shown in Fig. 2B, HFD elevated the mRNA levels of several pro-inflammatory biomarkers, whereas celastrol diminished the mRNA expression of pro-inflammatory genes including IL-6, IL-1β, TNF-α and iNOS, and also marginally increased the mRNA level of IL-10 over arginase-1. On the other hand, hepatic tissues were stained with antibodies against iNOS, COX-2 and arginase-1, whereas hepatic macro-phages were stained with anti-CD68 antibody and the cell nuclei were stained with DAPI. As shown in Fig. 2C, HFD increased the expression of iNOS and COX-2 in hepatic macrophages, whereas celastrol treatment selectively reduced iNOS expression over COX-2. Interestingly, celastrol enhanced arginase-1 expression in hepatic macrophages. Furthermore, the expression of inflammation and macrophage biomarkers was determined by qRT-PCR technique. As shown in Fig. 2D, qRT-PCR revealed that HFD generally increased the mRNA expression of selected biomarkers for inflammation and macrophage polarization. However, celastrol at the dose of 7.5 mg/kg/d effectively reduced the mRNA expression of pro-inflammatory genes including IL-6, IL-1β, TNF-α and iNOS back to the normal levels or even lower levels, while upregulated the expression level of arginase-1 mRNA and to a lesser extent, IL-10 mRNA.

### Celastrol suppressed proinflammatory M1 macrophage polarization in murine macrophage RAW264.7 cells

**Figure 3:**
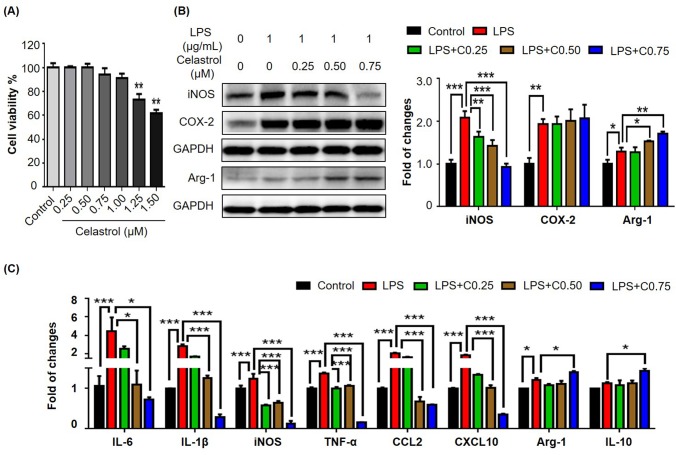
Effects of celastrol on the expression of macrophage M1 and M2 biomarkers in RAW264.7 cells (**A**) Cytotoxicity of celastrol. Following 48 h treatment, RAW264.7 cells were determined for the cell viability by a colorimetric MTT assay relative to the untreated controls (n = 3). **, *p* < 0.01 (Celastrol vs Control). (**B**) Effects of celastrol on the expression of iNOS, COX‐2 and arginase‐1 in LPS‐stimulated RAW264.7 cells. After 24 h treatment with LPS and/or celastrol, the cellular proteins were analyzed by Western blotting with specific antibodies and GAPDH (as loading control). The blots (n = 3) were quantified by a densitometric method. Representative blots were shown. (**C**) qRT‐PCR quantification of macrophage M1 and M2 biomarkers in RAW264.7 cells. After treatment with LPS and/or celastrol, the mRNA levels of specific biomarkers (n = 3) were analyzed by qRT‐PCR technique using Qiagen primers. C0.25, celastrol (0.25 μM); C0.5, celastrol (0.5 μM); C0.75, celastrol (0.75 μM); *, *p* < 0.05; **, *p* < 0.01; ***, *p* < 0.001.

To clarify whether celastrol targets macrophages, we examined the effects of celastrol on the expression of selected biomarkers in mouse macrophage cell line RAW264.7. We firstly evaluated the effect of celastrol on the cell viability in RAW264.7 cells over a period of 48 h. As shown in Fig. 3A, celastrol could exhibit detectable cytotoxicity when the concentration was raised up to 1.25 μM (*p* < 0.01). Celastrol eventually exhibited the IC_50_ value of 1.69 μM against the growth of RAW264.7 cells. We thereby treated RAW264.7 cells with celastrol at the concentrations below 1.25 μM for other experiments. We subsequently investigated the effects of celastrol on the expression of iNOS, COX-2 and arginase-1 by Western blot analysis. RAW264.7 cells were pre-treated with celastrol for 1 h and challenged with LPS for another 24 h. As shown in Fig. 3B, celastrol remarkably attenuated LPS-induced iNOS expression (*p* < 0.001), effectively up-regulated arginase-1 expression (*p* < 0.01) in a concentration-dependent manner. Unexpectedly, celastrol appeared to potentiate LPS-induced COX-2 expression. We further investigated the effects of celastrol on the mRNA expression of several key pro-inflammatory M1 and anti-inflammatory M2 biomarkers by qRT-PCR technique. As shown in Fig. 3C, LPS profoundly up-regulated six common M1 macrophage biomarkers while showed less effects on the expression of M2 macrophage biomarkers arginase-1 and IL-10. Celastrol not only suppressed LPS-induced expression of M1 macrophage biomarkers including IL-6, IL-1β, iNOS, TNF-α, CCL2 and CXCL-10 in a concentration-dependent manner, but also upregulated the mRNA expression of M2 macrophage biomarkers arginase-1 and IL-10 (*p* < 0.05) when the concentration was raised to 0.75 μM.

### Celastrol attenuated LPS-induced activation of MAP kinases in macrophages

**Figure 4:**
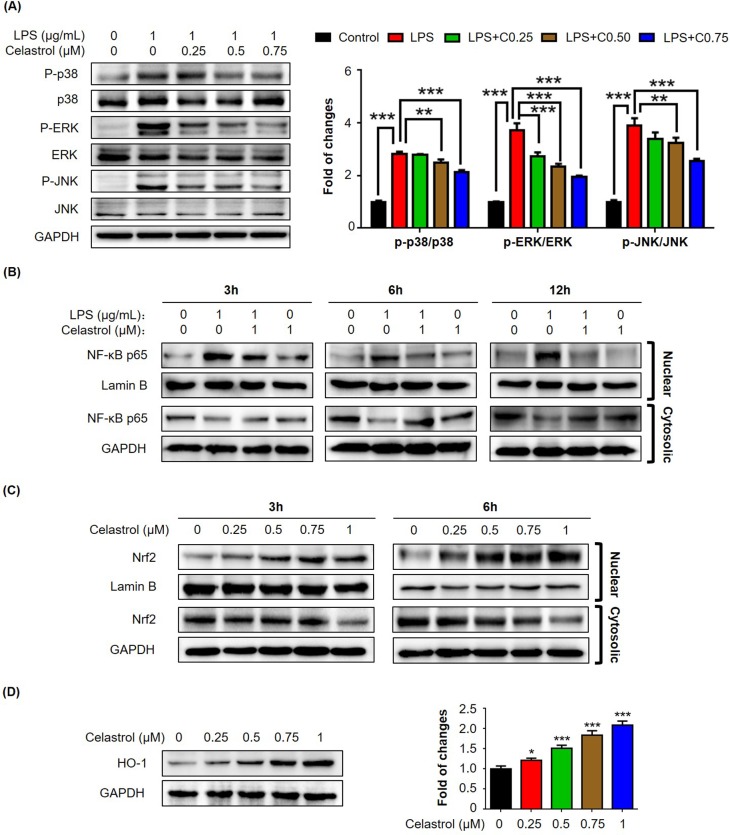
Effects of celastrol on the activation of MAP kinases, NF-κB and Nrf2/HO-1 pathways in RAW264.7 cells (**A**) Western blot analysis of MAP kinase activation in LPS-stimulated RAW264.7 cells. After treatment with LPS alone or in combination with celastrol, the cellular proteins were analyzed by Western blotting with specific antibodies. Representative blots were shown. The blots (n = 3) were quantified by a densitometric method. C0.25, celastrol (0.25 μM); C0.5, celastrol (0.5 μM); C0.75, celastrol (0.75 μM); **, *p* < 0.01; ***, *p* < 0.001. (**B**) Nuclear translocation of NF-κB p65 subunit. After treatment with 1 μg/ml LPS alone or in combination with 1 μM celastrol, the nuclear and cytoplasmic proteins were prepared and analyzed by Western blotting with specific antibodies. Lamin B and GAPDH were used as loading control. Representative blots were shown. (**C**) Nuclear translocation of Nrf2. After treatment with celastrol, the nuclear and cytoplasmic proteins were prepared and analyzed by Western blotting with specific antibodies. Lamin B and GAPDH were used as loading control. Representative blots were shown. (**D**) Induction of HO-1 expression. After 24 h treatment with celastrol, the cellular proteins were analyzed by Western blotting with specific antibodies and GAPDH (as loading control). Representative blots were shown. The blots (n = 3) were quantified by a densitometric method. *, *p* < 0.05; ***, *p* < 0.001.

To elucidate the mechanisms by which celastrol regulates macrophage polarization, we firstly examined the effects of celastrol on LPS-induced activation of MAP kinases in RAW264.7 cells. We pretreated the cells with celastrol at the concentrations of 0.25, 0.5 and 0.75 μM for 1 h and challenged with LPS for another 24 h. The activation of MAP kinases was examined by Western blot analysis. As shown in Fig. 4A, LPS (1 μg/mL) dramatically activated the phosphorylation of p38, extracellular signal-regulated kinase (ERK1/2) and JNK. Celastrol effectively attenuated LPS-induced phosphorylation of p38, ERK1/2 and JNK in a concentration-dependent manner.

### Celastrol inhibited LPS-induced nuclear translocation of NF-κB p65 but activated Nrf2/HO-1 pathway

To further investigate the effects of celastrol on pro-inflammatory NF-κB pathway and antioxidant Nrf2/HO-1 pathway, we focused on the nuclear translocation of transcriptional factors NF-κB p65 subunit and Nrf2 and the induction of anti-oxidant enzyme HO-1. For the nuclear translocation of NF-κB p65 subunit, we pretreated the cells with 1 μM celastrol for 1 h and subsequently challenged the cells with LPS for another 3, 6 or 12 h. Nuclear and cytoplasmic proteins were prepared and analyzed by Western blot analysis. As shown in Fig. 4B, LPS profoundly increased the presence of NF-κB p65 subunit in the nuclear fractions. Cleastrol effectively attenuated the stimulatory effects of LPS on the nuclear translocation of NF-κB p65 subunit and promoted the sequestration of NF-κB p65 subunit in the cytosol in a time-dependent manner. On the other hand, for the nuclear translocation of Nrf2, we treated the cells with 0.25, 0.5, 0.75 and 1 μM celastrol for 3 or 6 h. Nuclear and cytoplasmic proteins were similarly prepared and analyzed by Western blot analysis. As shown in Fig. 4C, celastrol effectively induced the nuclear translocation of Nrf2 in a concentration- and time-dependent manner. Furthermore, we treated the cells with celastrol for 24 h and examined the expression levels of Nrf2-target gene HO-1 by Western blot analysis. As shown in Fig. 4D, celastrol indeed induced HO-1 expression in a concentration-dependent manner.

### Celastrol suppressed macrophage M1 polarization via Nrf2/HO-1-mediated mechanism

**Figure 5:**
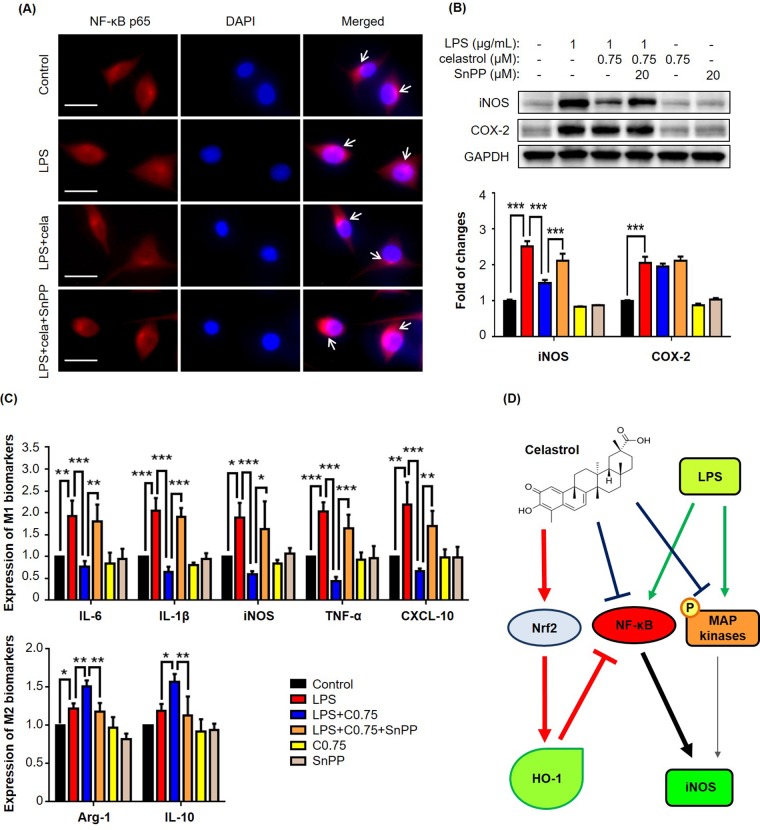
HO-1 inhibitor SnPP attenuated NF-κB activation and macrophage biomarker expression against celastrol in LPS-stimulated RAW264.7 cells (**A**) Restoration of NF-κB nuclear translocation against celastrol in LPS-stimulated macrophages. After drug treatment, the cells were stained for NF-κB p65 subunit, whereas the cell nuclei were stained with DAPI. The cells were imaged under a Zeiss fluorescence microscopy. Representative images were shown. Cela, celastrol (0.75 μM). Scale bar, 20 μm. (**B**) Specific up-regulation of iNOS expression against celastrol in LPS-stimulated macrophages. After 24 h treatment, the cellular proteins were analyzed by Western blotting with specific antibodies and GAPDH (as loading control). The blots (n = 3) were quantified by a densitometric method. Representative blots were shown. ***, *p* < 0.001. (**C**) Attenuation of celastrol inhibition on macrophage M1 and M2 biomarkers in LPS-stimulated RAW264.7 cells. After drug treatment, the expression of selected macrophage M1 and M2 biomarkers was quantified by qRT-PCR technique using Qiagen primers. C0.75, celastrol (0.75 μM); *, *p* < 0.05; **, *p* < 0.01; ***, *p* < 0.001. (**D**) Schematic illustration of the potential mechanisms. Celastrol activates Nrf2/HO-1 pathway while inhibits MAP kinases (e.g., ERK1/2, p38, JNK) and NF-κB pathway.

To clarify whether celatrol stimulates the phenotypic switch of macrophages via inducing HO-1 expression, we conducted three different experiments to examine the effects of HO-1 inhibitor Sn(IV) protoporphyrin IX dichloride (SnPP) on the nuclear translocation of NF-κB p65 subunit, expression of iNOS and COX-2, and expression of macrophage biomarkers. For Experiment 1, we treated RAW264.7 cells with LPS, celastrol and specific HO-1 inhibitor SnPP, alone or in combination, for 6 h. We subsequently detected the intracellular localization of NF-κB p65 subunit by immunofluores- cence staining. As shown in Fig. 5A, HO-1 inhibitor SnPP effectively abolished the inhibitory effect of celastrol on LPS-induced nuclear translocation of NF-κB p65 subunit. For Experiment 2, we treated RAW264.7 cells with LPS, celastrol and specific HO-1 inhibitor SnPP, alone or in combination, for 24 h. The protein levels of iNOS and COX-2 were analyzed by Western blotting. As shown in Fig. 5B, celastrol attenuated LPS-induced iNOS expression, whereas HO-1 inhibitor SnPP eventually abolished the activity of celastrol against LPS-induced iNOS expression. Neither of celastrol and SnPP affected COX-2 expression in LPS-stimulated macrophages. For Experiment 3, we treated RAW264.7 cells with LPS, celastrol and specific HO-1 inhibitor SnPP, alone or in combination, for 24 h. We determined the effects of HO-1 inhibitor on the mRNA levels of macrophage M1 and M2 biomarkers relative to LPS and celastrol. As shown in Fig. 5C, HO-1 inhibitor SnPP diminished the activities of celastrol against LPS-induced mRNA expression of the pro-inflammatory M1 biomarkers (e.g., IL-6, IL-1β, iNOS and TNF-α) and the anti-inflammatory M2 biomarkers (e.g., arginase-1 and IL-10).

## DISCUSSION

Macrophage phenotypes are associated with adipose tissue inflammation, metabolic disorders and insulin resistance in obesity [24, 25]. Inflammatory M1 macrophages cause adipose tissue inflammation and insulin resistance, whereas anti-inflammatory M2 macrophages ameliorate obesity-induced insulin resis-tance [26]. The aim of the present study was to examine whether plant-derived celastrol could promote weight loss and attenuate inflammation in diet-induced obese mice via suppressing macrophage M1 polarization while stimulating macrophage M2 polarization. We confirmed that celastrol not only effectively promoted weight loss but also improved glucose tolerance and insulin sensitivity. In the present study, we focused on the effects of celastrol on the expression of macrophage polarization biomarkers and the underlying mechanisms.

Celastrol was proven to be effective in the control of weight gain and blood glucose levels in mouse and rats models of high fat-induced obesity [19, 22]. As far as the drug safety is concerned, celastrol did not cause evident toxic effect in wild-type mice after i.p. adminis-tration of 100 μg/kg/d for 195 and 216 days [19]. In the present study, we firstly validated the effects of celastrol on weight gain in obese mice (Fig. 1A-B). Although celastrol was administered at the dose of up to 7.5 mg/kg/d by oral gavage, no sign of toxicity was observed. Consistently, it was previously demonstrated that celastrol at the dose of 10 mg/kg/d did not cause weight loss in lean mice over three weeks of treatment [19]. By examining the effects of celastrol on glucose tolerance and insulin insensitivity, we found that celastrol could ameliorate obesity-disrupted glucose homeostasis and insulin resistance (Fig. 1C-D). As far as the pathology of obesity, low-grade chronic inflammation is a direct cause for insulin resistance and metabolic dysfunctions in adipose tissues [7]. The inflamed adipose tissues secrete various inflammatory signals to recruit macrophages. Adipose tissue infiltrating macrophages were proinflammatory and exhibited classically activated M1 phenotype [27]. Based on the previous study on the tissue distribution of celastrol [28], after i.p. administration of 4 mg/kg for a period of 240 min, the prolonged and steady levels of celastrol were detected in liver but not in other organs from KM mice. In the present study, therefore, we focused on the effects of celastrol on macrophage M1/M2 polarization in livers and adipose tissues. We employed Western blotting, qRT-PCR and fluorescence immunostaining to determine the expression levels of typical macrophage M1 and M2 biomarkers. By examining the adipose tissues and livers from the experimental mice, we found more M1 macrophages in mice on high fat diet, while M2 macrophages in mice on high fat diet supplemented with celastrol. Moreover, celastrol at the dose of 7.5 mg/kg/d decreased the expression of pro-inflammatory M1 biomarkers while increased the expression of macrophage M2 biomarkers (e.g., arginase-1 and IL-10) in adipose tissues and livers (Fig. 2). In addition, we also found that celastrol decreased the plasma levels of IL-6 and IL-1β in HFD-fed mice (Fig. 1E-F), which was consistent with the previous findings [29]. Taken together, our results suggested that celastrol suppressed macrophage M1 polarization, but somewhat enhanced macrophage M2 polarization against *in vivo* inflammation in adipose tissues and livers.

MAP kinases (e.g., ERK1/2, JNK and p38) and NF-κB signaling pathway are implicated in obesity-induced type-2 diabetes and insulin resistance [30]. It was found that MAP kinases and NF-κB pathway were activated in multiple tissues in obesity, possibly promoting tissue inflammation [31]. Other studies discovered that pro-inflammatory cytokines and free fatty acids caused the over-activation of JNK, leading to the insulin resistance and diabetes [14]. Along this line, celastrol was previously demonstrated to inhibit the activation of MAP kinases and NF-κB pathway, and thereby decrease the release of inflammatory cytokines [32]. Moreover, high-fat meal increased circulating LPS levels in type 2 diabetic patients [33]. Animal studies showed that HFD enhanced circulating LPS levels through altering intestinal permeability [27]. The same study also demonstrated that subcutaneous infusion of LPS could initiate obesity and insulin resistance. In the present, therefore, LPS was used to activate macrophage RAW264.7 cells. Indeed, we found that LPS increased the expression of a bunch of pro-inflammatory M1 macrophage biomarkers. Interestingly, our results revealed that celastrol down-regulated the mRNA and protein levels of LPS-induced M1 biomarkers (e.g., IL-6, IL-1β, iNOS and TNF-α) while enhanced the mRNA and protein levels of anti-inflammatory M2 markers (e.g., arginase-1 and IL-10) in a concentration-dependent manner (Fig. 3). Moreover, the present study demonstrated that celastrol antagonized LPS activity to activate MAP kinases and NF-κB pathway (Fig. 4A-B). In addition, a recent study showed that celastrol could induce HO-1 expression through reactive oxygen species (ROS)/Nrf2/ARE mechanisms [34]. In the present study, we firstly confirmed that celastrol stimulated the nuclear translocation of Nrf2 and induced HO-1 expression in a concentration dependent manner (Fig. 4C-D). We subsequently clarified that HO-1 inhibitor SnPP could largely diminish the inhibitory effects of celastrol on LPS-induced activation of NF-κB pathway and the synthesis of macrophage M1 and M2 biomarkers by immunofluorescence staining, western blot analysis and qRT-PCR technique (Fig.5A-C). Collectively our results suggested that celastrol might suppress macrophage M1 polarization against inflam-mation through two related mechanisms: direct induction of HO-1 expression through inhibiting the activation of MAP kinases and NF-κB pathway and activating Nrf2/HO-1 pathway (Fig. 5D).

In summary, the present study validated that celastrol not only promoted the weight loss but also ameliorated glucose tolerance and insulin insensitivity in diet-induced obese mice. The key finding was that the anti-inflammatory and anti-obesity activities of celastrol may be linked by promoting macrophage polarization from M1 to M2 phenotype. Such information warrants further investigations on the potential roles of macro-phage polarization in hepatic and adipose tissues in the regulation of leptin activity and appetize in the brain. Nevertheless, our results support the development of celastrol as a promising drug candidate for treating obesity.

## MATERIALS AND METHODS

### Antibodies and biochemical reagents

Antibodies against p38, ERK1/2, JNK, GAPDH, Lamin B, phospho-p38, phospho-ERK1/2, phospho-JNK, COX-2 were purchased from Cell Signaling Technology (Boston, MA, USA). Antibodies against arginase-1, HO-1, Nrf2 and NF-κB p65 were purchased from Santa Cruz Biotechnology (Dallas, TX, USA). Antibodies against iNOS and CD68 were purchased from Abcam (Cambridge, UK). Anti-rabbit HRP-conjugated IgG secondary antibody and lipopolysaccharide (LPS) were purchased from Sigma-Aldrich (St. Louis, MO, USA). Dulbecco's modified Eagle's Medium (DMEM), fetal bovine serum (FBS) and 100x penicillin and streptomycin solution were purchased from Invitrogen (Carlsbad, CA, USA). Protein assay dye reagent concentrate was purchased from Bio-Rad (Hercules, CA, USA). HO-1 inhibitor SnPP was purchased from Frontier Scientific Inc (Logan, UT, USA). Celastrol with the purity of over 98% by HPLC was purchased from Nanjing Spring and Autumn Biological Engineering Co., Ltd. (Nanjing, China).

### Mouse husbandry and drug treatment

Protocols for animal experiments were approved by the University of Hong Kong Committee on the Use of Live Animals in Teaching and Research (CULATR NO: 3755-15). For high fat diet-induced obese model, male C57BL/6N mice (age, 3-4 weeks; body weight, 11-13g) were fed on 60 kcal% HFD (Research Diets, Inc, New Brunswick, NJ, USA) for 12 weeks. Control mice were maintained on chow diet (13.5% from fat calories) (Lab Diet, Inc, St. Louis, MO, USA). Animals were housed under 12 h of light and 12 h dark cycle with free access to food and water at the Laboratory Animal Unit, University of Hong Kong. Celastrol were freshly prepared every day by dissolving the drug powder in saline containing 5% DMSO and 1% Tween-20. For drug treatment, celastrol was administrated to mice at the dose of 5 mg/kg/d or 7.5 mg/kg/d via oral gavage for consecutive 21 days. The mice in Control group and HFD group received the same volume of saline containing 5% DMSO and 1% Tween-20 by oral gavage. Body weights of mice were monitored on a daily basis.

### NMR determination of fat mass

Animal body fat composition was assessed using a benchtop Bruker minispec LF90 TD-NMR analyzer (Bruker Optics, Inc, Billerica, MA, USA) after celastrol treatment for 21 days essentially as previously described [19]. In brief, the minispec instrument was firstly calibrated daily using Bruker standards. Mice were weighted and then placed into the instrument for non-invasive examination.

### Assays of glucose tolerance test and insulin tolerance test

Blood glucose levels were measured by collecting blood from mice tail vein for assaying glucose tolerance and insulin sensitivity as described [19]. ipGTT was conducted in mice after 16 hours of fasting (18:00 p.m.-10:00a.m.). The mice were initially injected D-glucose at the dose of 1 g/kg. Blood was collected from mouse tail vein at 0, 15, 30, 60, 90, 120 min and measured for blood glucose levels using glucose test paper. On the other hand, ITT was conducted in mice after 6 hours of starvation (10:00 a.m.-16:00 p.m.). The mice were initially injected insulin at the dose of 0.75 IU/kg. Blood glucose levels were measured in the same manner as for ipGTT.

### Cell culture

Murine macrophage cell line RAW264.7 was obtained from the American Type Culture Collection (Manassas, VA, USA). The cells were cultured in DMEM supplemented with 10% FBS, and 1% penicillin/strep-tomycin (Invitrogen, CA, USA) at 37°C in a humidified incubator containing 5% CO_2_. For drug treatment, the cells were treated with indicated drugs as previously described [35].

### Measurement of cell viability

The cell viability was evaluated by a standard colorimetric assay using 3-[4, 5-dimethylthiazol-2-yl]-2, 5-diphenyltetrazolium bromide (MTT) as previously described [36]. In brief, the cells were treated with celastrol at the indicated concentrations for 48 h, and subsequently incubated with 0.5 mg/ml MTT in phosphate-buffered saline (PBS) for 4 h. Formazan pro-duction was determined by measuring the absorbance at 570 nm on a Bio-Rad microplate reader (Hercules, CA, USA). The cell viability was presented as a percentage relative to vehicle-treated controls.

### Quantitative real-time PCR determination of mRNA expression

The expression of biomarker mRNAs was determined by quantitative real-time PCR technique as described [37]. Briefly, total RNAs were isolated from the freshly collected livers and epididymal adipose tissues using TRIzol reagent (Invitrogen, CA, USA), and converted to the corresponding cDNAs using RevertAid first-strand cDNA synthesis kit (Thermo Fisher, Waltham, MA, USA). Quantitative real time-PCR was performed with specific primers from QIAGEN (Valencia, CA, USA) and detection reagent SYBR Green mix (QIAGEN, Valencia, CA, USA). The primers were obtained from QIAGEN and their assay numbers were as follows:

IL-6 (Mm_ll6_1_SG; QT00098875), Il-1β (Mm_ll1b_2_SG; QT01048355), iNOS (Mm_Nos2_1_SG; QT00100275), TNF-α (Mm_Tnf_1_SG; QT00104006), Arg-1 (Mm_Arg1_1_SG; QT00134288), IL-10 (Mm_ll10ra_1_SG; QT00112742), CCL-2 (Mm_Ccl2_1_SG; QT00167832), CXCL10 (Mm_Cxcl10_1_SG; QT00093436), GAPDH (Mm_Gapdh_3_SG; QT01658692). Gene-specific PCR products were subjected to melting curve analysis and quantified by the 2-ΔΔCt method whereas GAPDH mRNA was determined as an internal control.

### ELISA

The plasma levels of IL-6, TNF-α and IL-1β were measured by commercial ELISA kits (i.e., ab222503, ab208348 and ab100704) from Abcam (Cambridge, UK) according to the manufacturer's instructions. Mouse plasma was isolated from mouse blood in heparinized tubes by centrifuging at 3000 × g for 15 min at 4°C. The plasma samples were stored at −80°C before use.

### Extraction of total protein, nuclear and cytosolic protein

The total cellular, cytoplasmic and nuclear proteins were prepared as previously described [38]. After drug treatment, the cells were lysed in RIPA buffer (Sigma-Aldrich, St. Louis, MO, USA) with 1% (v/v) protease inhibitor cocktail for 30 min on ice. The cell lysates were centrifuged at 13000g for 15 min at 4°C. The supernatants were collected as the total cellular proteins. For the preparation of nuclear and cytosolic proteins, cells were washed and lysed with hypotonic buffer (20 mM Hepes (pH 8.0), 10 mM KCl, 1 mM EDTA, 1.5 mM MgCl_2_, 1 mM DTT, 1 mM Na_3_VO_4_, 1 mM NaF, 1 mM PMSF and 1% (v/v) protease inhibitor cocktail) on ice for 30 min. After adding 0.625% NP-40, cell lysates were centrifuged at 13000g for 15 min. The supernatants were collected as the cytosolic proteins, whereas the nuclear pellets were re-suspended in cold buffer (20 mM Hepes (pH 8.0), 1 mM EDTA, 1.5 mM MgCl_2_, 10 mM KCl, 1 mM DTT, 1 mM Na_3_VO_4_, 1 mM NaF, 1 mM PMSF, 1% (v/v) protease inhibitor cocktail and 20% (v/v) glycerol) for another 30 min. After centrifugation at 15000g for 5 min, the supernatants were collected as the nuclear extract. Protein concentration was determined using a Bio-Rad protein assay kit (Hercules, CA, USA).

### Western blot analysis

The protein levels of specific biomarkers were analyzed by Western blot analysis as described [37, 39]. In brief, thirty micrograms of the protein samples were resolved by 10% SDS-polyacrylamide gel electrophoresis and subsequently transferred to polyvinylidene difluoride (PVDF) membrane. Following 1-hour incubation in 5% non-fat milk powder or bovine serum albumin (BSA), the membranes were probed with primary antibodies against specific biomarkers overnight. The bound antibodies were detected with a goat anti-rabbit IgG-HRP conjugate. The blots were visualized by enhanced chemiluminescence (ECL) detection reagents from GE Healthcare (Uppsala, Sweden) under a Bio-Rad GelDoc imaging system (Hercules, CA, USA). The gel images were analyzed by NIH ImageJ software (http://imagej.net/ImageJ2).

### Immunofluorescence staining

The expression of specific biomarkers in mouse livers and epididymal adipose tissues was examined by immunofluorescence staining as previously described [39, 40]. Briefly, after drug treatment for 21 days, mice were sacrificed. Livers and epididymal adipose tissues were collected and fixed in 10% formalin in phosphate buffer at room temperature for at least 72 h. The fixed tissues were sequentially dehydrated with ethanol and xylene, embedded in paraffin and dissected into 5 μm tissue sections. For immunofluorescence detection, tissue sections were firstly revitalized in retrieval buffer (PH 6.0), permeabilized with 0.5% Triton X-100 for 30 min, and blocked with 5% normal goat serum in PBS for 2 h at room temperature. Section slides were then probed with specific primary antibodies overnight at 4°C, and subsequently detected by the secondary antibodies (i.e., Alexa Fluor 594-conjugated goat anti-rabbit IgG secondary antibody and Alexa Fluor 488-conjugated goat anti-mouse IgG secondary antibody) for 2 h at room temperature. The cell nuclei were stained with DAPI. After the removal of excessive fluorescence reagents, the liver and adipose tissues were imaged on a Zeiss LSM 780 confocal microscopy (Carl-Zeiss, Jena, Germany).

### Statistical analysis

The results were presented as mean ± SEM for the measurement of body weight and mean ± SD for the rest experiments. The differences between two groups were analyzed by one-way analysis of variance (ANOVA) with post hoc Dunnett's test using GraphPad Prism software (La Jolla, CA, USA). The *p*-values less than 0.05 were considered as significantly different.
